# Synthesis, characterization, and crystallization behaviors of poly(_D_-lactic acid)-based triblock copolymer

**DOI:** 10.1038/s41598-020-60458-9

**Published:** 2020-02-27

**Authors:** Yifan Wu, Lingtong Li, Shaopeng Chen, Jun Qin, Xiaolang Chen, Dengfeng Zhou, Hong Wu

**Affiliations:** 1 Key Laboratory of Advanced Materials Technology Ministry of Education, School of Materials Science and Engineering, Southwest Jiaotong University, Chengdu, 610031 China; 2 The State Key Laboratory of Polymer Materials Engineering, Polymer Research Institute of Sichuan University, Chengdu, 610065 China; 3 Key Laboratory of Karst Environment and Geohazard, Ministry of Land and Resources, Guizhou University, Guiyang, 550025 China; 4 Key Laboratory of Light Metal Materials Processing Technology of Guizhou Province, Guizhou Institute of Technology, Guiyang, 550003 China

**Keywords:** Polymers, Chemical physics

## Abstract

Poly(_D_-lactic acid) (PDLA) with different polyethylene glycol (PEG) segment synthesized PDLA-PEG-PDLA triblock copolymer through the ring-opening reaction of _D_-LA and PEG will be used as a toughening modifier. The microstructure, crystal structures and crystallization behaviors of this triblock copolymer were investigated by Fourier transform infrared (FTIR) spectroscopy, nuclear magnetic resonance (NMR) spectroscopy, X-ray diffraction (XRD), differential scanning calorimetry (DSC) and polarized optical microscopy (POM). The triblock copolymer is synthesized from the appearance of CH_2_ stretching vibration peak at 2910 cm^−1^ and C-O stretching vibration peak at 1200 cm^−1^ from PEG in FTIR spectra. Moreover, the chemical shift that is about 3.6 ppm in ^1^H NMR and 68.8ppm in ^13^C NMR proves this matter. The results of XRD and DSC reveal that PDLA and PEG are crystallized separately, and are not fully compatible, and microphase separation has occurred in this triblock copolymer. PEG can induce the triblock copolymer to accelerate the rate of crystallization, allowing it to crystallize more completely in the same amount of time. When the molecular weight of PEG is 6000 or the ratio of _D_-LA/PEG is 1/1, the crystallizability of PDLA-PEG-PDLA triblock copolymer is the best.

## Introduction

The rapid development of industry has brought great convenience to people’s lives, but the environmental pollution caused by the rapid development is also gradually exposed, coupled with the oil crisis caused by the extensive development and utilization of petroleum resources, the necessity and urgency of the research and development of degradable polymers are gradually highlighted. Among the various degradable polymers, polylactic acid (PLA) is the most promising as a green polymer material^1–3^. The biggest and most prominent advantage of PLA is that it can be recycled in nature^4^. Its raw materials are renewable biological resources, so it is no longer dependent on petroleum and other resources. Its wastes are completely transformed into carbon dioxide and water through hydrolysis and a series of biological metabolism, which is harmless to human body and non-toxic, and has no pollution to the environment^5,6^.

According to the chiral property of the structural unit, PLA can be divided into poly (_L_-lactic acid) (PLLA), poly (_D_-lactic acid) (PDLA) and poly (_D_, _L_-lactic acid) (PDLLA)^7^. However, compared with polyesters with higher rigidity, PLA is a semi-crystalline polymer, which usually has a slower crystallization rate without external force and produces some amorphous products during processing^8,9^. And its heat resistance is poor, which limits its application requirements in bioengineering, food packaging and other industries^10–12^.

Currently, in order to improve its physical or thermodynamic properties, PLA is modified by adding other substances to form composites (such as rice husk hydrochar^13^, lignin^14^, polycarbonate^15^, chitosan^16^, etc.) and polymerizing with other substances (such as PLA-PCVL-PLA^17^, SEBS-g-PLA^18^, etc.). Of course, there are also some investigations on the crystallization behavior of PLA. For example, Zhang *et al*.^19^ adds lactide-caprolactone copolymer (LACL) into polylactide/poly (ε-caprolactone) (PLA/PCL) blends. PCL reduces the spherulite size of crystallization of PLA, and the nuclear density PLA/PCL/LACL blends is far higher than that of PLA and PLA/PCL. The results indicate that LACL has a capacity-enhancing effect on immiscible PLA/PCL blends, thus promoting the nucleation of PLA. The crystallization half-life of PLA/PCL/LACL and PLA/PCL is also lower than that of pure PLA, and the crystallization rate of the blends increases. Similarly, PLA, cellulose nanofibers (CNFs) and glycidyl methacrylate (GMA) grafted poly (lactic acid) (PLA-g-GMA) are mixed to fabricate PLA/CNFs and PLA/PLA-g-GMA/CNFs composites^20^. When 0.1 wt% CNFs are added, the crystallization rate of the composites is improved markedly. What’s more, the combination of plasticizer and nucleating agent (dioctyl adipate and ethylene bis stearic amide) can effectively improve the crystallization rate of PLA^21^.

Using external conditions to enhance the crystallization ability of PLA is the current research hotspot. Wang *et al*.^22^ investigates the isothermal crystallization kinetics of a series of long-chain polylactic acid induced by shear, and finds that the crystallization of PLA with different degree of branched is significantly improved under shear, and the crystal morphology also changes from spherulite to oriented crystallization. The properties of PLA are controlled by the laser flame, which induces the polymer to increase its crystallinity at lower temperatures and mainly forms α′ crystalline structures^23^.

Polyethylene glycol (PEG), a water-soluble polyether achieved by the progressive addition of ethylene oxide to water or ethylene glycol, has good molecular chain flexibility, non-toxicity and good biocompatibility^24^. It was found that PEG grafted onto the surface of starch resulted in PEG enriched areas around starch particles, thus forming a new interfacial transition layer between PLA and starch, so the elongation at break and impact strength of the blend could be improved^25^. However, there are few studies on the effect of PEG on the crystallization behavior of PLA.

In this work, PDLA-PEG-PDLA triblock copolymer with different PEG molecular weight and the ratio of _D_-LA/PEG was prepared by ion exchange method. PEG, as the second component, was involved in the polymerization reaction, which changed the microstructure of the copolymer. The effect of different PEG molecular weight and the ratio of _D_-LA/PEG on the microstructure, crystal structure and crystallization behaviors of copolymer was studied by Fourier transform infrared (FTIR) spectroscopy, nuclear magnetic resonance spectroscopy (NMR), X-ray diffraction (XRD), differential scanning calorimetry (DSC) and polarized optical microscopy (POM).

## Experimental

### Materials

Polyethylene glycol (PEG) (AR) was purchased from Chengdu Kelong Chemical Reagent Company (China), with molecular weights of 2000, 4000, 6000, and 8000. _D_-lactide (_D_-LA, 99%, Mw = 144.13) was acquired from Shanghai Macklin Biochemical Co. Ltd., China. Stannous octoate (Sn(Oct)_2_, 95%) was obtained from Shanghai Aladdin Biochemical Technology Co. Ltd., China. Dichloromethane (CH_2_Cl_2_) (AR), Petroleum ether (AR) and xylene (AR) was supplied by Chengdu Kelong Chemical Reagent Company (China).

### Synthesis of PDLA-PEG-PDLA triblock copolymers

**Figure 1:**
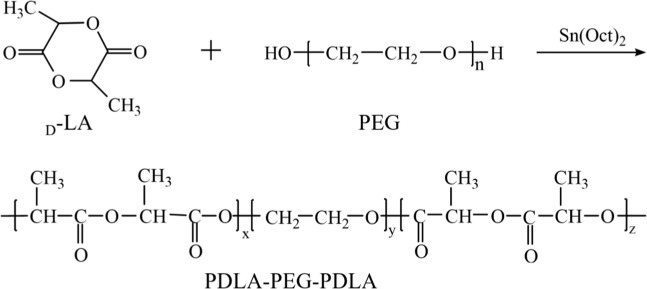
Synthesis of PDLA-PEG-PDLA triblock copolymer.

The PDLA-PEG-PDLA triblock copolymers were synthesized by ring-opening polymerization (ROP) of _D_-lactide using PEG as an initiator and stannous octoate as a catalyst^26^. The synthetic route is shown in Fig. 1, where n, x, y and z were the degree of polymerization of PEG part and PDLA part, respectively. Firstly, _D_-LA and PEG with different molecular weight were dried at 50 °C in an electric blast drying oven for 2 h. The required _D_-LA and PEG with different molecular weights were accurately weighed by an electronic balance, then the medicine was placed in a dry three-necked flask, and nitrogen was passed through. After 5 minutes, the air in the flask was completely removed and the three-necked flask was placed in an oil bath pot and heated at a constant temperature. The temperature of the oil bath was 140 °C and the magnetic stirring was 15 minutes until the drug was completely dissolved. Sn(Oct)_2_/xylene was injected into the mixed solution. Dosage of Sn(Oct)_2_ was about 0.6 wt% of _D_-LA. The reaction was stirred at 160 °C for 4 h under nitrogen atmosphere. After the reaction, the crude product was dissolved into CH_2_Cl_2_ to wash the unreacted drug. And then petroleum ether was used as precipitant to further purify the product and obtain pure PDLA-PEG-PDLA triblock copolymer after filtration. The obtained precipitates were dried under vacuum at room temperature for 24 h. When the ratio of _D_-LA/PEG was constant, a series of block copolymers were prepared by changing the molecular weight of PEG (2000, 4000, 6000, 8000). And when PEG molecular weight was fixed, a series of block copolymers were obtained by changing the ratio of _D_-LA/PEG (1/1, 2/1, 3/1).

### Measurements and characterization

#### Fourier transform infrared (FTIR) spectra

A Thermo Nicolet 560 FTIR spectrometer (USA) was applied to monitor the FTIR spectra of the samples in the range of 4000–400 cm^−1^. A small amount of fully dried sample to be tested and potassium bromide were ground evenly in a mortar, then placed on a tablet press and pressed into translucent flakes at a pressure of 10 Mpa. The flakes were tested in a Fourier infrared spectrometer.

#### Nuclear magnetic resonance (NMR) spectroscopy

The NMR resonance spectroscopy was carried out on a Swiss Bruker model AVANCE400 with tetramethylsilane as the internal standard in CDCl_3_ at 25 °C.

#### X-ray diffraction (XRD)

The crystal structure was measured by X-ray diffractometer (X’pert PRO, made by the Panalytical Company, Netherlands) with Cu-Kα radiation (*λ* = 0.15418) at 40 kV and 40 mA. The diffraction patterns were collected at 2*θ* between 5–60° at a scanning rate of 5°/min.

#### Differential scanning calorimetry (DSC)

DSC thermograms were obtained with a Q20 (TA Instruments, USA). About 8–10 mg specimens were heated from room temperature to 200 °C at the rate of 10 °C/min, maintained at 200 °C for 5 min to erase the thermal history. The samples after premelting were cooled to room temperature at a cooling rate of 10 °C/min, and then subsequently reheated to 200 °C at a rate of 10 °C/min, and the DSC curves were recorded. All the tests were conducted under nitrogen atmosphere.

#### Polarized optical microscopy (POM)

The POM technology was employed to investigate the crystal morphology on a CX40P polarizing microscope (Ningbo Shunyu Instrument Co., Ltd., China). The samples were placed on a hot table at 170 °C. After the samples were completely melted, they were pressed into thin sheets with cover glass, and then put into an incubator with a set temperature. After crystallization for a certain period of time, the samples were taken out for observation.

## Results and Discussion

### Characterization of PDLA-PEG-PDLA triblock copolymer

**Figure 2:**
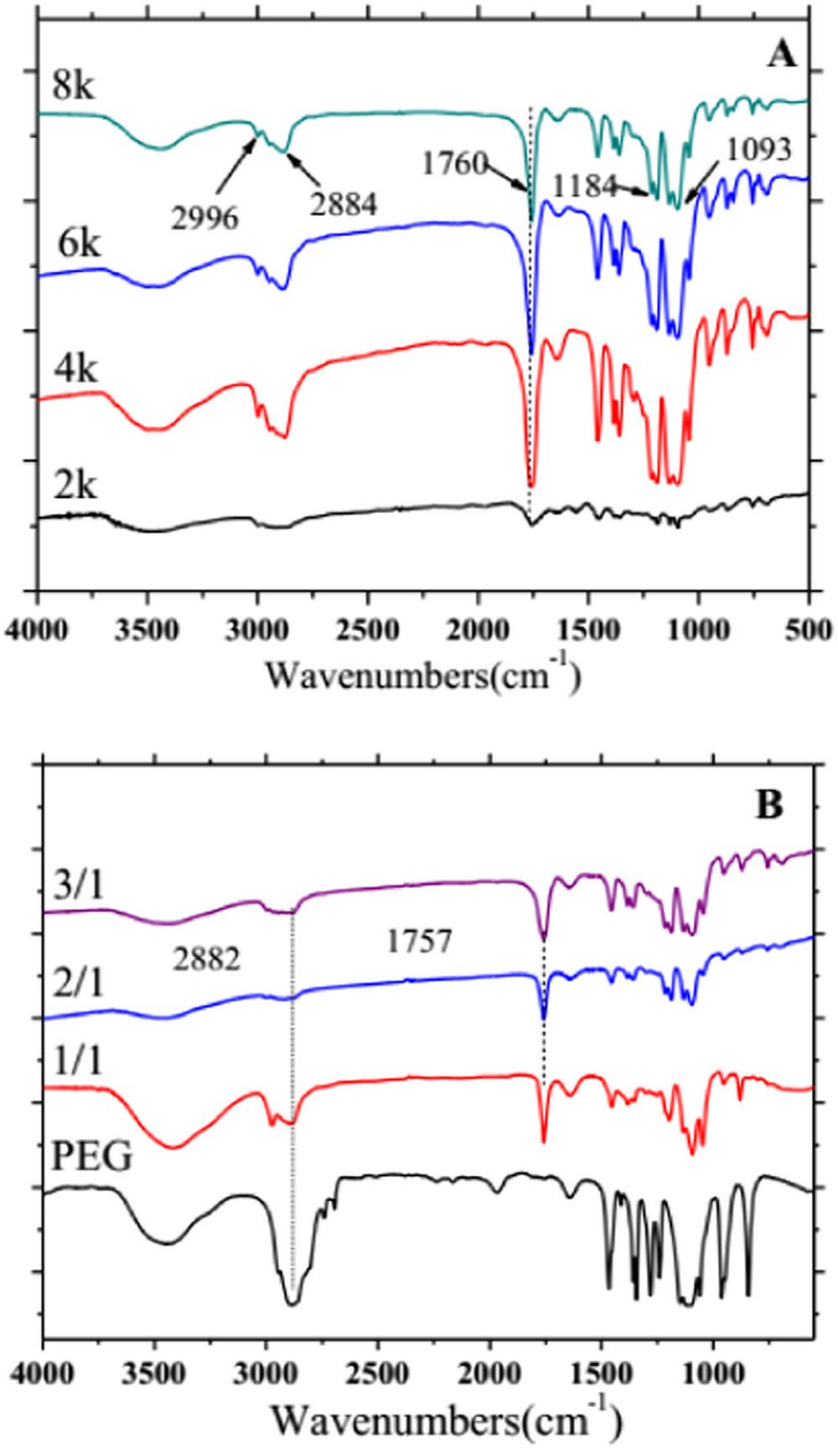
FTIR spectra of PDLA-PEG-PDLA triblock copolymers with different PEG segments: (**A**) PEG with different molecular weights is 2000, 4000, 6000, and 8000; (**B**) The ratio of _D_-LA/PEG is 1/1, 2/1, and 3/1, respectively.

In order to understand whether PDLA-PEG-PDLA triblock copolymer is successfully synthesized, the block copolymer samples synthesized with different segments of PEG were characterized by FTIR, as shown in Fig. 2. As can be seen that there is a strong absorption peak at 3400 cm^−1^, corresponding to the alcoholic hydroxyl group at the end of the block copolymer^27^. The two absorption peaks at 2990 and 1600 cm^−1^ is due to the stretching vibration of CH_3_ and the antisymmetric bending vibration, respectively, which prove the existence of CH_3_ functional group^28^. 2910 and 2890 cm^−1^ can be observed in this figure, the two closely spaced absorption peaks represent the absorption peaks of CH and CH_2_, respectively. And in the fingerprint region, there is a 1460 cm^−1^ bending vibration peak of CH_2_ and in the range of 1350 to 1310 cm^−1^ of CH bending vibration peaks. These absorption peaks prove the introduction of PEG structure in PDLA, and the presence of methyl and hypomethyl groups from PDLA and methylene groups from PEG in molecular chains, indicating that PDLA successfully copolymerized with PEG. The stretching vibration peak of C=O appears at 1760 cm^−1^, and the intensity of the peak is strong. Since it is connected with oxygen atoms, the electron absorption induction effect is greater than the conjugate effect of electron donor, which makes the absorption peak of the whole carbonyl group shift to the region of high wavenumber. And this is a big proof of the existence of ester groups. The peaks in the range from 1300 to 1200 cm^−1^ are strong, corresponding to the stretching vibration peak of C-O, and the absorption peaks close to 1120 cm^−1^, 1100 cm^−1^ and 1000 cm^−1^ is the anti-symmetric stretching vibration peak of C-O-C. From the above analysis, the conclusion that PDLA-PEG-PDLA block copolymer was synthesized successfully can be obtained preliminarily.

**Figure 3:**
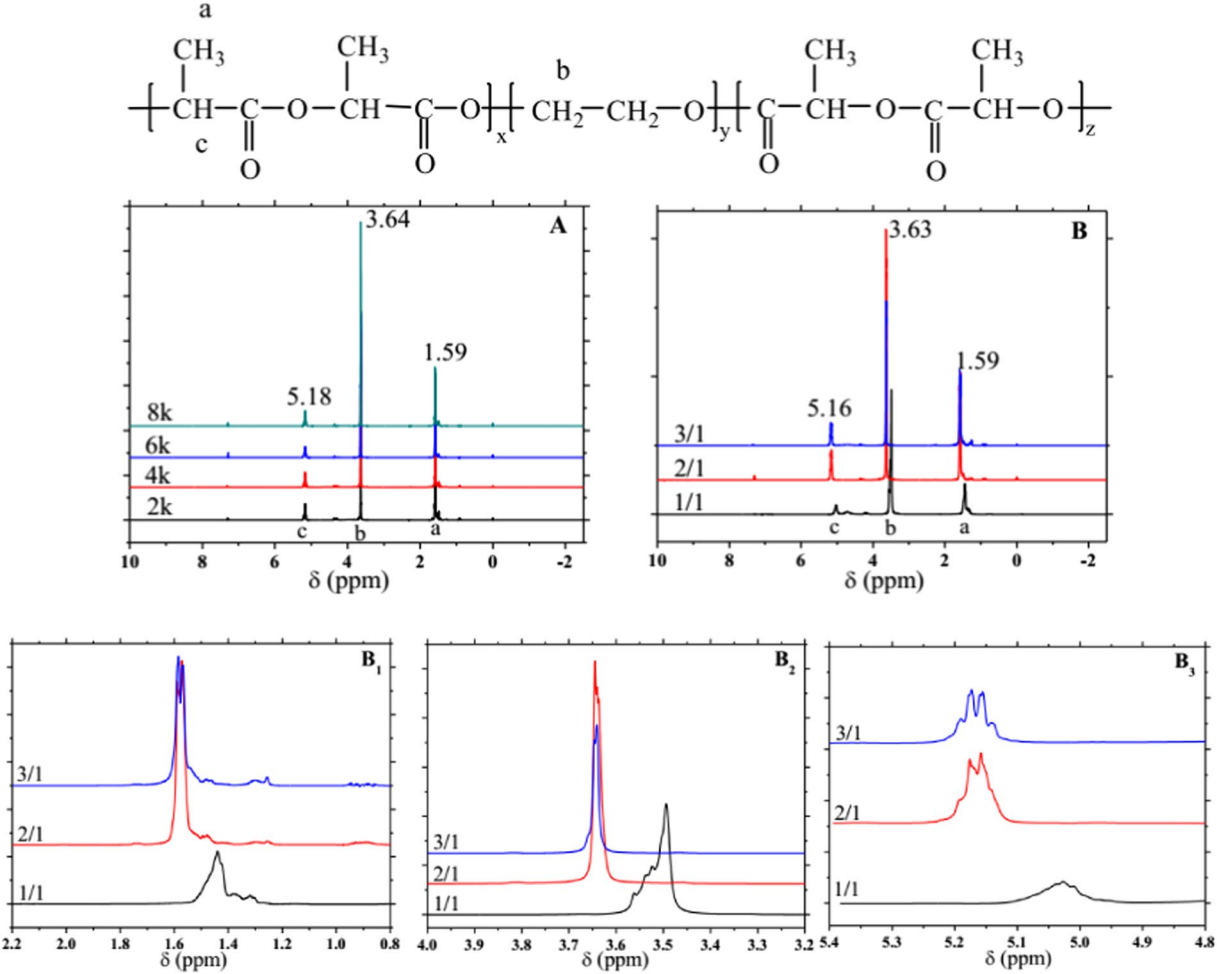
^1^H NMR spectra of PDLA-PEG-PDLA triblock copolymers with different PEG segments. (**A**) PEG with different molecular weights is 2000, 4000, 6000, and 8000. (**B**) The ratio of _D_-LA/PEG is 1/1, 2/1, and 3/1, respectively; (**B**_**1**_) 2.5–0.7 ppm of B; (**B**_**2**_) 4.3–3.2 ppm of B and (**B**_**3**_) 5.35–4.8 ppm of B.

The composition of the synthesized PDLA-PEG-PDLA triblock copolymer was determined by NMR spectroscopy, as shown in Fig. 3 (^1^H NMR) and 4 (^13^C NMR). Nuclear magnetic resonance (NMR) spectroscopy, which studies the absorption of radiofrequency radiation by atomic nuclei, is one of the most powerful tools for qualitative analysis of the composition and structure of a variety of organic and inorganic things^29^. In the ^1^H NMR, there are three groups of peaks, indicating that H atom in this copolymer has three chemical environments, and each group of peaks of different copolymers is roughly in the same position in both Fig. 3A,B. The chemical shift of methine (CH) and methyl (CH_3_) protons in PDLA is observed around 5.15–5.20 ppm (peak c) and 1.60 ppm (peak a), respectively. The methylene protons in (CH_2_) group of PEG are around 3.63 ppm (peak b). When D-LA: PEG is 1/1, the chemical displacement of absorption peak moves in the direction of decreasing in Fig. 3B. This is because the electron absorption capacity of C=O decreases, which increases the electron cloud density around the hydrogen nucleus, and the shielding effect increases. Therefore, the resonance absorption peak moves to the high field, and the chemical displacement decreases.

**Figure 4:**
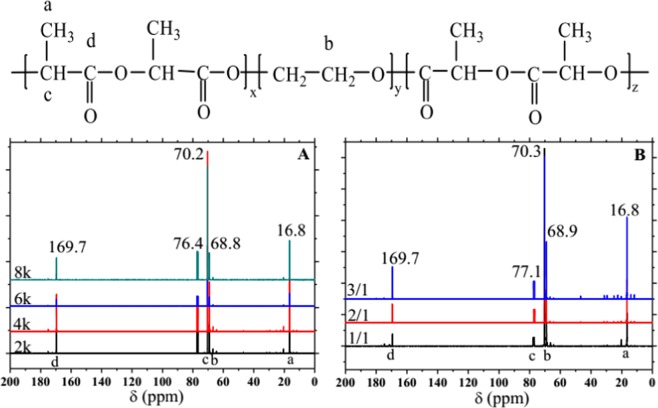
^13^C NMR spectra of PDLA-PEG-PDLA triblock copolymers with different PEG segments: (**A**) PEG with different molecular weights is 2000, 4000, 6000, and 8000 and (**B**) The ratio of _D_-LA/PEG is 1/1, 2/1, and 3/1, respectively.

Figure 4 is the ^13^C NMR of PDLA-PEG-PDLA triblock copolymer. Among the isotopes of C, only ^13^C has spin phenomenon, and nuclear magnetic resonance absorption. The principle is the same as that of the hydrogen spectrum, but the chemical displacement range of the carbon spectrum is very wide, generally 0~250 ppm^30^. Even if the chemical environment difference of C is very small, the peak can be separated in the carbon spectrum. The positions of absorption peaks in Fig. 4A,B are roughly the same. The chemical shift of the absorption in the spectrum at 16.8 ppm (peak a) is the carbon atom of CH_3_ from PDLA; the chemical shift at 68.8 ppm (peak b) is the carbon atom of CH_2_ from PEG; the chemical shift peak at 70.2 ppm (peak c) is the carbon atom of CH from PDLA and the chemical shift at 169.7 ppm (peak d) is the carbon atom from PDLA. The absorption peak of tritium chloroform was 76–77 ppm. So far, combined with the FTIR which has been analyzed before, it is a strong demonstration that the PDLA-PEG-PDLA block copolymer has been successfully synthesized.

### Crystal structure of PDLA-PEG-PDLA triblock copolymer

**Figure 5:**
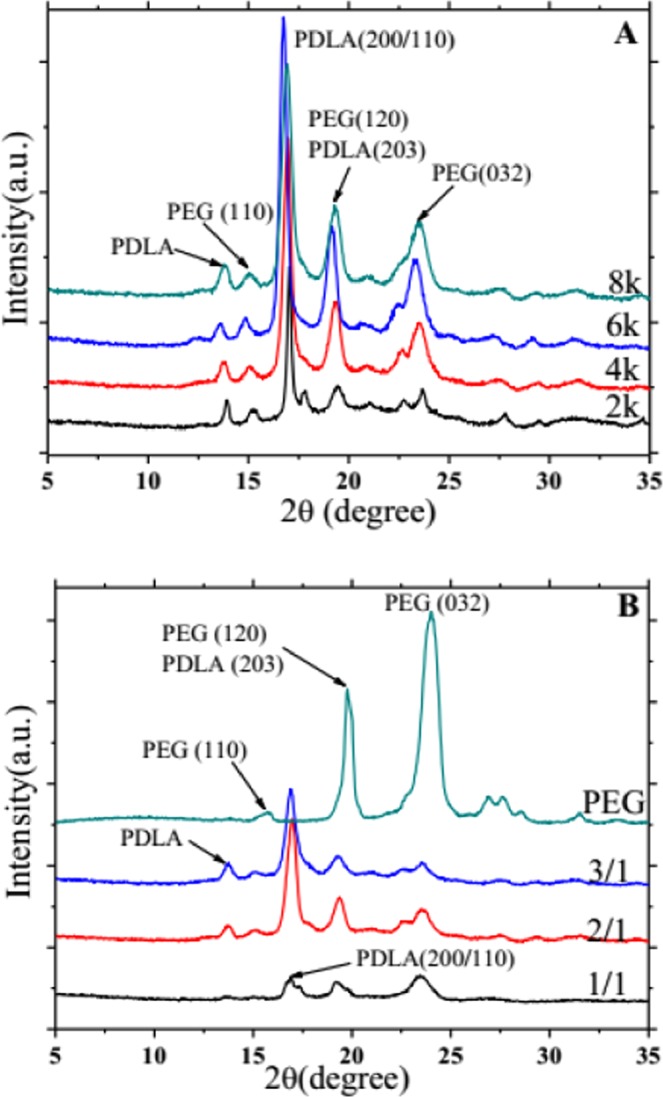
X-ray diffraction spectra of PDLA-PEG-PDLA triblock copolymers with different PEG segments: (**A**) PEG with different molecular weights is 2000, 4000, 6000, and 8000; (**B**) The ratio of _D_-LA/PEG is 1/1, 2/1, and 3/1, respectively.

XRD analysis can effectively provide the crystal structure information of polymer. Figure 5 shows the XRD patterns PDLA-PEG-PDLA triblock copolymer with different lengths of PEG segments. In Fig. 5A, a sharp diffraction peak appears at 2θ is 16.8°, and the corresponding crystal lattice index is (200)/(110) of α-type of PLA structure^31^. Diffraction peaks appear at 19.3° and 23.5°, corresponding to (120) and (032) crystal plane diffraction of PEG, respectively^32^. PDLA also has diffraction peaks at 19.3° is (203) crystal plane. There are also some weak diffraction peaks at 13.9° and 15.1° belonging to PDLA and PEG, respectively. The positions of the respective diffraction peaks are substantially the same, and as the molecular weight of PEG from 2000 to 6000 is increased, the intensity of the diffraction peak is also increased, but when the molecular weight is too large, reaching 8000, the strength is weakened. It may be that the over-molecular weight of PEG limits the diffusion of the molecular chain of the block copolymer, thereby limiting crystallization. When the molecular weight of PEG is 6000, the intensity of the diffraction peak of PDLA reaches the maximum, however, the position of the peak remains unchanged. The change of the diffraction peak of PEG is not obvious. The results reveal that the effect of different molecular weight of PEG on the crystallization of block copolymer is not significant when the content of PEG is fixed, and the crystal form does not change. It may be that there is little difference in the molecular chain size of PEG with different molecular weights. There is no significant difference in the molecular chain movement during crystallization, and the structure formed is the same. Only the changes in the diffraction peak intensity are shown in the XRD pattern. However, when the content of PEG in block copolymer is different, the structure of sample is obviously different.

In Fig. 5B, the diffraction peaks at 13.9°, 15.1°, 19.3°, and 23.5° are still present, but the intensity of the diffraction peaks is different due to the difference in _D_-LA: PEG ratio. When the content of PDLA is low (1/1), the diffraction peak at 13.9° does not appear, and the diffraction peak of PEG is dominant. With more of PDLA, the characteristic peak (16.8°) strength of PDLA gradually increases, while the diffraction peak of PEG gradually weakens. This further demonstrates that PEG induces the crystallization of the copolymer. At the same time, PEG and PDLA exhibit respective crystal diffraction peaks, meaning that the two blocks are not completely compatible in the copolymer and present a state of microphase separation.

### Crystallization and melting behaviors of PDLA-PEG-PDLA triblock copolymer

**Figure 6:**
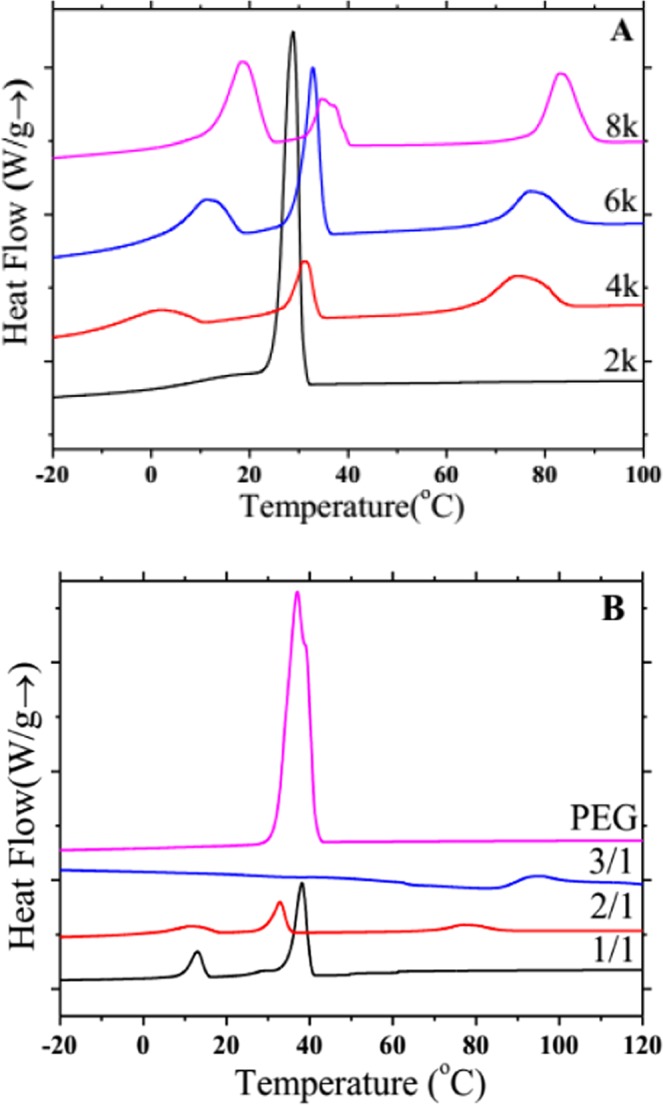
DSC crystallization curves of PDLA-PEG-PDLA triblock copolymers with different PEG segments at a cooling rate of 10 °C/min: (**A**) PEG with different molecular weights is 2000, 4000, 6000, and 8000; (**B**) PEG and _D_-LA/PEG with various ratios of 1/1, 2/1, and 3/1.

**Figure 7:**
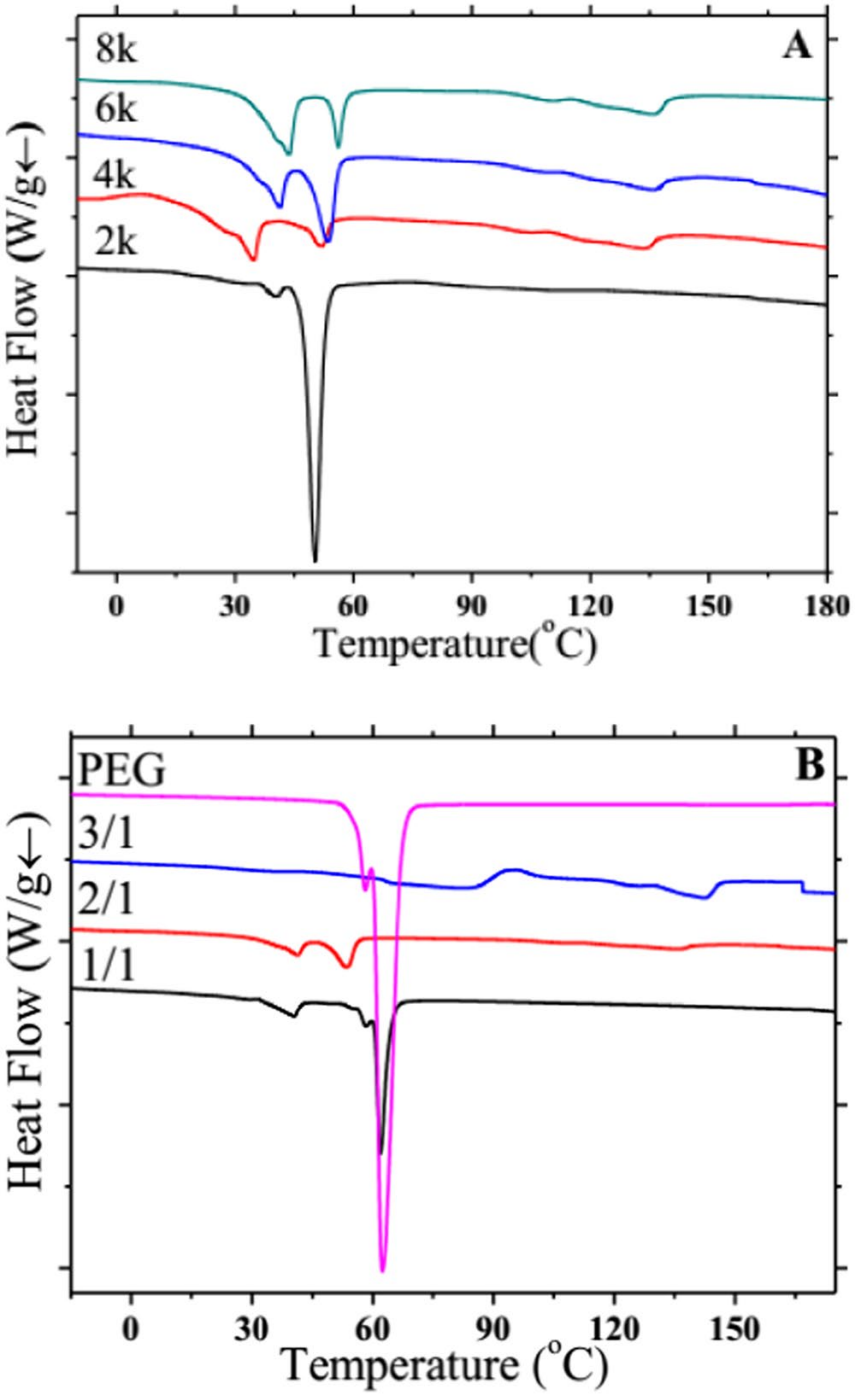
DSC melting curves of PDLA-PEG-PDLA triblock copolymers with different PEG segments at a heating rate of 10 °C/min: (**A**) PEG with different molecular weights is 2000, 4000, 6000, and 8000; (**B**) PEG and _D_-LA/PEG with various ratios of 1/1, 2/1, and 3/1.

Figures 6 and 7 show the DSC crystallization and melting curves of PDLA-PEG-PDLA triblock copolymers with different PEG segments and various ratios of _D_-LA/PEG, and the corresponding DSC detailed data are listed in Tables 1 and 2, respectively. The exothermic peak of low temperature (such as 28.8 and 31.1 °C) is the crystallization peak of PEG, and the exothermic peak at 80–90 °C is the crystallization peak of PDLA. When the molecular weight of PEG is too low (2000), there is no crystallization peak of PDLA in the DSC curves, only the crystallization peak of PEG. Low molecular weight PEG has no prominent effect on promoting the crystallization of block copolymer, and the molecular chain of low molecular weight PEG does not make the molecular chain of copolymer to be more flexible. Moreover, when the molecular weight of PEG is increased from 2000 to 4000, the crystallization peak of PEG reduces significantly, and the crystallization peak of PDLA also appears. The molecular weight of PEG continues to increase, and the crystallization temperatures of PEG and PDLA also increase slightly. In Fig. 6B, when the content of PEG is high, we only found the crystallization peak of PEG. As the PDLA content increases, the crystallization peak of PDLA also arises, and the crystallization temperature of PDLA is remarkably improved. Thus, the addition of a certain amount of PEG can induce the crystallization of block copolymers, which may be because PEG as a soft segment improves the mobility of copolymers, thus making them easier to crystallize. Too high PEG content or too large PEG molecular weight may entangle the molecular chains of the block copolymer. Too low content or too small PEG molecular weight does not significantly increase the flexibility of the molecular chains and does not promote the crystallization of the block copolymer. And the change of PEG content in copolymer has a greater influence on the crystallization of block copolymer than the change of PEG molecular weight. The appropriate PEG content increases the molecular chain flexibility of the copolymer and avoids entanglement, which makes it easy to move in the crystallization process and form more crystals. The DSC melting curves expresses the same rule, as shown in Fig. 7. When the PEG molecular weight is 2000 or the PEG content is higher, only the PEG melting peak is found, and the PEG molecular weight increases or the PEG content decreases, and the melt peak tends to weaken. From the results of DSC, it is observed that the two segments of the block copolymer retain their own independent individuals with their respective crystallization and melting peaks. This is also consistent with the results of XRD, suggesting that PDLA and PEG are in a state of microphase separation.

**Table 1:** DSC results for PDLA-PEG-PDLA triblock copolymers with different molecular weights of PEG segments.

Samples	*T*_c,PEG_ (°C)	Δ*H*_c,PEG_ (J/g)	*T*_m,PEG_ (°C)	Δ*H*_m,PEG_ (J/g)	*T*_c,PDLA_(°C)	Δ*H*_c,PDLA_ (J/g)	*T*_m,PDLA_ (°C)	Δ*H*_m,PDLA_ (J/g)
PEG2k	28.8	28.8	50.3	25.8	—	—	—	—
PEG4k	1.5 31.1	10.0	34.8 52.2	10.1	74.4	6.6	133.6	2.7
PEG6k	11.1 32.8	19.3	41.3 53.5	15.1	76.9	7.5	135.8	3.3
PEG8k	18.4 34.3	17.0	43.7 54.2	13.8	82.9	10.3	135.8	10.4

PEG with different molecular weights is 2000, 4000, 6000, and 8000, respectively.

**Table 2:** DSC results for PEG and PDLA-PEG-PDLA triblock copolymers with different proportion of _D_-LA: PEG.

Samples	*T*_c,PEG_ (°C)	Δ*H*_c,PEG_ (J/g)	*T*_m,PEG_ (°C)	Δ*H*_m,PEG_ (J/g)	*T*_c,PDLA_(°C)	Δ*H*_c,PDLA_ (J/g)	*T*_m,PDLA_ (°C)	Δ*H*_m,PDLA_ (J/g)
PEG	37.0	161.9	62.4	164.1	—	—	—	—
1/1	12.9 38.1	46.4	40.5 61.8	37.1	—	—	—	—
2/1	11.1 32.8	19.3	41.3 53.5	15.2	76.9	7.5	135.8	3.3
3/1	—	—	—	—	94.3	12.9	142.7	12.7

The ratio of _D_-LA/PEG is 1/1, 2/1, and 3/1, respectively.

### Crystal morphology of PDLA-PEG-PDLA triblock copolymer

**Figure 8:**
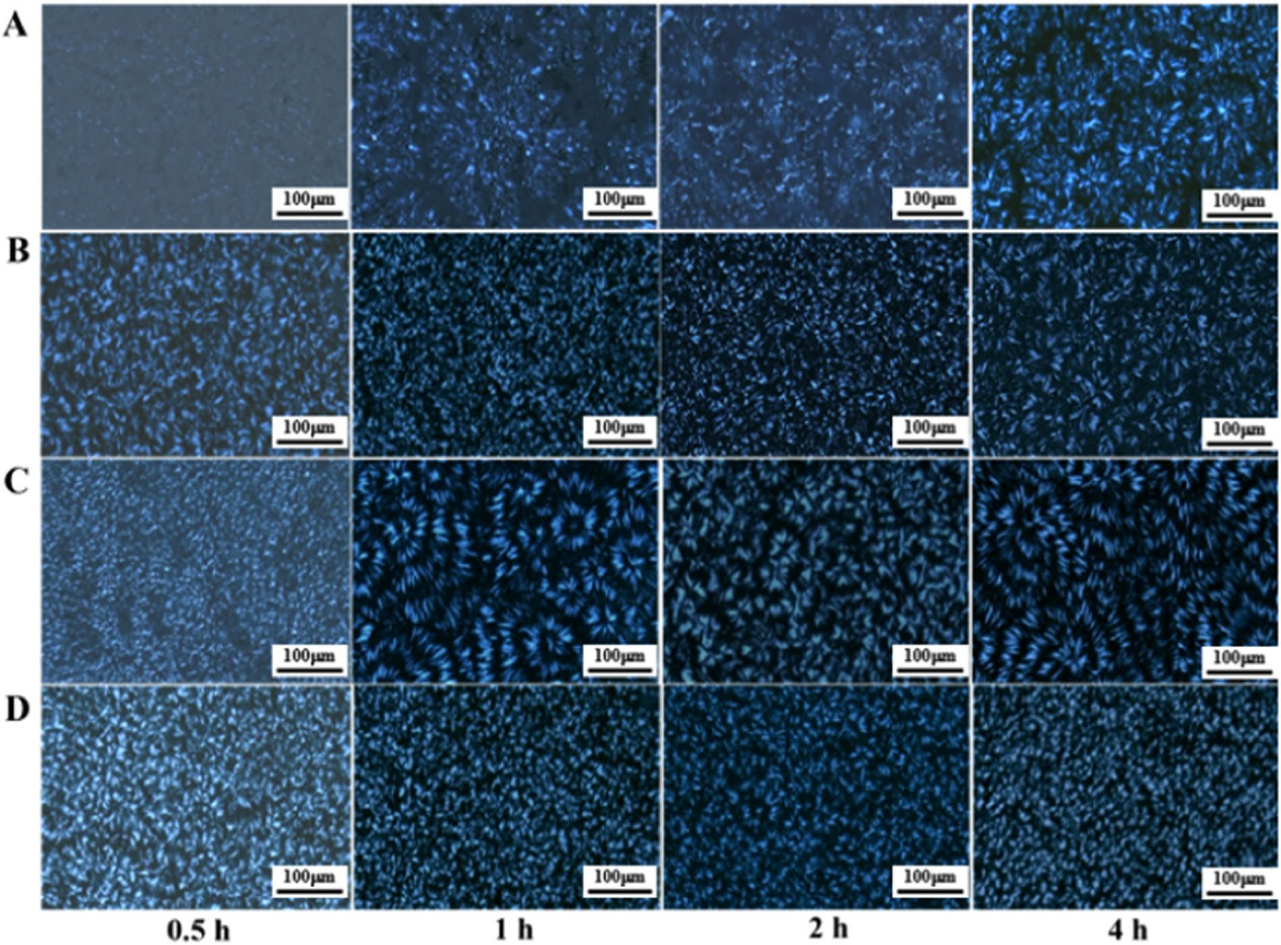
POM micrographs of PDLA-PEG-PDLA triblock copolymers with different PEG segments at 100 °C during the isothermal crystallization process. PEG with different molecular weights is 2000 (**A**), 4000 (**B**), 6000 (**C**), and 8000 (**D**).

Through POM, the effects of different holding times on the crystallization behavior of PDLA-PEG-PDLA triblock copolymers with different PEG segments can be visually observed. Figure 8 shows POM micrographs of PDLA-PEG-PDLA triblock copolymers with different PEG segments at 100 °C for different time (0.5, 1, 2, and 4 h). The triblock copolymer can crystallize at 100 °C, but the difference in molecular weight of PEG has a great influence on the crystallization behavior. When the holding time is the same, the spherulite size increases with the increase of PEG molecular weight, but when the molecular weight reaches 8000, the spherulite size decreases. And no matter what the molecular weight of PEG is, the crystallization temperature remains unchanged, the crystallization becomes more perfect and the spherulite growth is complete with the extension of the insulation time. At the right temperature and holding time, PDLA chain segments have enough movement time and movement ability. PEG as the soft segment is introduced to reduce the force between and within PDLA macromolecules, weaken the hydrogen bond between molecules, and improve the activity and softness of molecular chains, which can be quickly arranged into the lattice^33^. It is clear that with the increase of PEG molecular weight, copolymer molecular chains become softer, crystallize faster and have larger spherulites.

**Figure 9:**
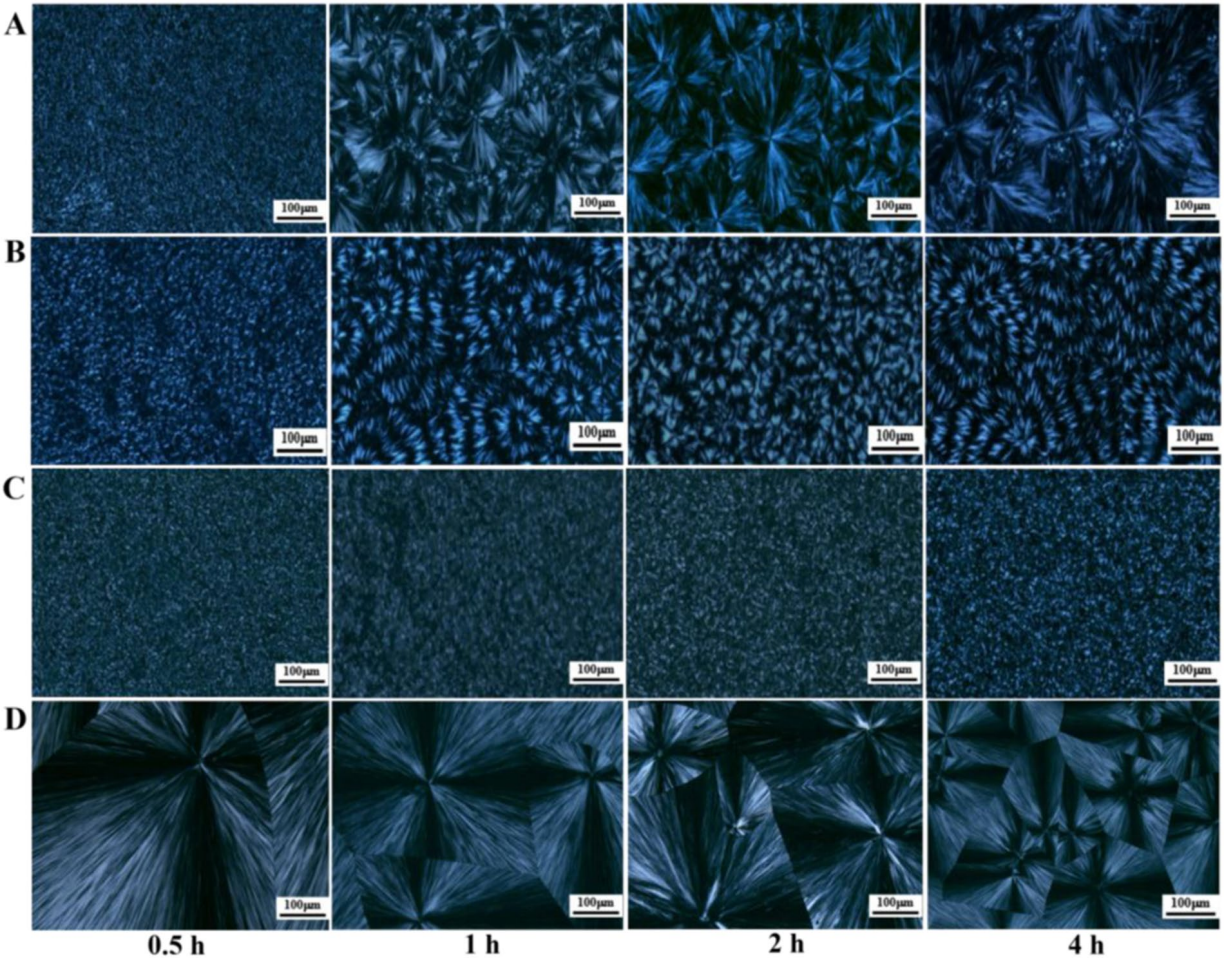
POM micrographs of PEG and PDLA-PEG-PDLA triblock copolymers with different block ratio at 100 °C during the isothermal crystallization process. _D_-LA/PEG with various ratios of 1/1 (**A**), 2/1 (**B**), 3/1 (**C**), and PEG (**D**).

Figure 9 shows the POM micrographs of PEG6000 and PDLA-PEG-PDLA triblock copolymers with different ratios of _D_-LA: PEG at 100 °C during the isothermal crystallization process for different time (0.5, 1, 2, and 4 h). The crystallization of PEG is very rapid, and the spherulite size is large. But the spherulite density is low. We find only one spherulite, filling the field of vision, and even the part near the nucleus can be seen at 100 °C for 0.5 h. As the holding time is prolonged, more crystals grow and the density of spherulites increases. As for copolymers, in general, the larger the PEG content, the larger the spherulite size at the same time. Moreover, the change in the block ratio is more pronounced than the change in the molecular weight of the PEG on the crystallization behavior of the copolymer. This also fully demonstrates that PEG increases the mobility of molecular chains of block copolymers and induces their crystallization to some extent.

## Conclusions

PDLA-PEG-PDLA triblock copolymers are successfully synthesized by solution method. The FTIR and NMR results exhibit the characteristic peaks of CH_2_ and C-O, chemical displacement of protons and carbon atoms, respectively, confirming that the copolymerization has already taken place, and PEG segment is introduced into PDLA successfully. In the XRD pattern, PDLA and PEG have their own diffraction peaks. When the molecular weight of PEG is changed, there is no large change in the position and intensity of the diffraction peak. But when the PEG content is large (1/1), the diffraction peak of PDLA is weak. The crystallization peak and melting peak of PDLA and PEG also appear separately in the crystallization and melting curves of DSC. This is similar to the results of XRD, indicating that PDLA and PEG are not completely compatible and present a state of microphase separation. The POM results reveal that the addition of PEG allows the copolymer to form crystals faster and with a larger grain size. This may be PEG as a soft segment, which enhances the flexibility and fluidity of the molecular chain and promotes the crystallization of PDLA. In addition, compared with the change of PEG molecular weight, the difference of PEG content has a greater effect on crystallization behavior.
